# MetaGenyo: a web tool for meta-analysis of genetic association studies

**DOI:** 10.1186/s12859-017-1990-4

**Published:** 2017-12-16

**Authors:** Jordi Martorell-Marugan, Daniel Toro-Dominguez, Marta E. Alarcon-Riquelme, Pedro Carmona-Saez

**Affiliations:** 1 Bioinformatics Unit, Centre for Genomics and Oncological Research (GENYO), Granada, Spain; 2 Medical Genomics, Centre for Genomics and Oncological Research (GENYO), Granada, Spain; 3 Institute for Environmental Medicine, Karolinska Institutet, Stockholm, Sweden

**Keywords:** Genetic association study, Meta-analysis, Web tool, Shiny

## Abstract

**Background:**

Genetic association studies (GAS) aims to evaluate the association between genetic variants and phenotypes. In the last few years, the number of this type of study has increased exponentially, but the results are not always reproducible due to experimental designs, low sample sizes and other methodological errors. In this field, meta-analysis techniques are becoming very popular tools to combine results across studies to increase statistical power and to resolve discrepancies in genetic association studies. A meta-analysis summarizes research findings, increases statistical power and enables the identification of genuine associations between genotypes and phenotypes. Meta-analysis techniques are increasingly used in GAS, but it is also increasing the amount of published meta-analysis containing different errors. Although there are several software packages that implement meta-analysis, none of them are specifically designed for genetic association studies and in most cases their use requires advanced programming or scripting expertise.

**Results:**

We have developed MetaGenyo, a web tool for meta-analysis in GAS. MetaGenyo implements a complete and comprehensive workflow that can be executed in an easy-to-use environment without programming knowledge. MetaGenyo has been developed to guide users through the main steps of a GAS meta-analysis, covering Hardy-Weinberg test, statistical association for different genetic models, analysis of heterogeneity, testing for publication bias, subgroup analysis and robustness testing of the results.

**Conclusions:**

MetaGenyo is a useful tool to conduct comprehensive genetic association meta-analysis. The application is freely available at http://bioinfo.genyo.es/metagenyo/.

**Electronic supplementary material:**

The online version of this article (10.1186/s12859-017-1990-4) contains supplementary material, which is available to authorized users.

## Background

Genetic association studies (GAS) estimate the statistical association between genetic variants and a given phenotype, usually complex diseases [1]. In the last few years, the number of genetic association studies has increased exponentially, but the results are not consistently reproducible. This lack of reproducibility may be influenced by several factors, including the analysis of non-heritable phenotype, inappropriate quality control, wrong statistical analysis, low sample size, population stratification, incorrect multiple-testing correction or technical biases [2].

Meta-analysis is a statistical technique for combining results across studies and it is becoming very popular as a method for resolving discrepancies in GAS. It summarizes research findings, increases statistical power and enables the identification of genuine associations [3]. In this context, in 2011 there was a 64-fold increase in genetics-related meta-analysis compared to 1995 [4].

Despite the increasing number of publications in this field there is a lack of dedicated software tools to perform a complete GAS meta-analysis in a friendly environment. In this context, most published works in the field have used commercial software suites such as STATA [5] or SPSS [6]. These are statistical software packages that include general functions for meta-analysis in their configuration. In addition, freely available R packages such as meta [7] or metafor [8] are also widely used but all these solutions share common limitations: do not provide all required steps for a GAS meta-analysis (e.g. evaluating Hardy Weinberg equilibrium (HWE) or genetic models) and require advanced statistical or bioinformatics knowledge to be properly used.

In this context, Park et al. have reported several analytical errors in published GAS meta-analysis [9], many of them could be avoided using a dedicated software for GAS meta-analysis with predefined functions and automatic computations of the required statistics.

Here we present MetaGenyo, an easy-to-use web application which implements a complete meta-analysis workflow for GAS. Once the data has been loaded, it provides a guided and complete workflow that comprises the main steps in GAS meta-analysis, including HWE test, checking heterogeneity, publication bias indicators, statistical association testing for different genetics models, subgroup analysis and robustness testing. The use of MetaGenyo does not require advanced statistical or bioinformatics knowledge and we hope it will be a useful application for researchers working in the field of genetic association studies.

## Implementation

MetaGenyo has been implemented as a web tool using shiny [10], a web application framework for RStudio [11]. Backend computations are carried out in R using available packages and custom scripts. MetaGenyo provides the following functionalities:

### Testing HWE

Departures from HWE can occur due to genotyping errors, selection bias and stratification [12]. Therefore, goodness-of-fit of HWE should be checked in each study before pooling data. HardyWeinberg package [13, 14] is used to compute a *P*-value for each study in the control population in order to identify low-quality studies. As we test for HWE in several studies, the obtained *P*-values are corrected by Benjamini and Hochberg false discovery rate (FDR) [15].

### Genetic models

Given two alleles (A, a) the three possible genotypes (AA, Aa, aa) can be dichotomized in different ways yielding different genetic models. GAS can be carried out assuming a specific genetic model based on biological criteria but in most of the cases different models are simultaneously evaluated. MetaGenyo performs meta-analysis in several ways [16], including allele contrast (A vs. a), recessive (AA vs. Aa + aa), dominant (AA + Aa vs. aa) and overdominant (Aa vs. AA + aa) genetic models as well as pairwise comparisons (AA vs. aa, AA vs. Aa and Aa vs. aa). All *P*-values are adjusted for multiple testing with the Bonferroni method [17].

### Statistical analysis and heterogeneity

To perform meta-analysis, MetaGenyo combines the effect sizes of the included studies by weighting the data according to the amount of information in each study. Association values are calculated based on two different statistic models: Fixed Effects Model (FEM) and Random Effects Model (REM). The choosing between both models depends on the amount of heterogeneity in the data, which is also evaluated with heterogeneity indicators such as I^2^ and Cochran’s Q test (see on-line help of the program). Meta package (7) is used to get such heterogeneity indicators and association results. Finally, this same package is used to generate forest plots to summarize information for effect size and the corresponding 95% confidence interval (CI) of each study and the pooled effect. Forest plots can be generated for FEM, REM or both, and can be downloaded with very high resolution.

### Publication bias

Publication bias occurs because of meta-analysis are performed using published studies, which usually report only significant associations, while studies showing no significant results tend to remain unpublished. This may therefore give a falsely skewed positive result. To test for publication bias, MetaGenyo provides funnel plots and Egger’s test [16] for each genetic model. Funnel plots are generated with meta package [7] and Egger’s test is performed using the metafor package [8].

### Subgroup analysis

MetaGenyo provides a subgroup analysis in order to evaluate associations in a subset of studies based on the user defined criteria (e.g. studies from the same country). Many genetic associations are population-specific and can be undiscovered in a general meta-analysis, but discovered when studies are split. For each group, a meta-analysis is performed with FEM or REM, depending on the heterogeneity test: If heterogeneity *P*-value <0.1, REM will be used. Otherwise, FEM will be used instead. These results are downloadable in Excel and text formats.

### Sensitivity analysis

In order to test the robustness of the meta-analysis performed, MetaGenyo performs a leave-one-out influence analysis using meta package [7]. Briefly, the meta-analysis is repeated several times, each time excluding one of the studies, in order to determine how each individual study affects the overall statistics [18]. A forest plot with these results is generated for the selected genetic model.

### Software usage

**Fig. 1:**
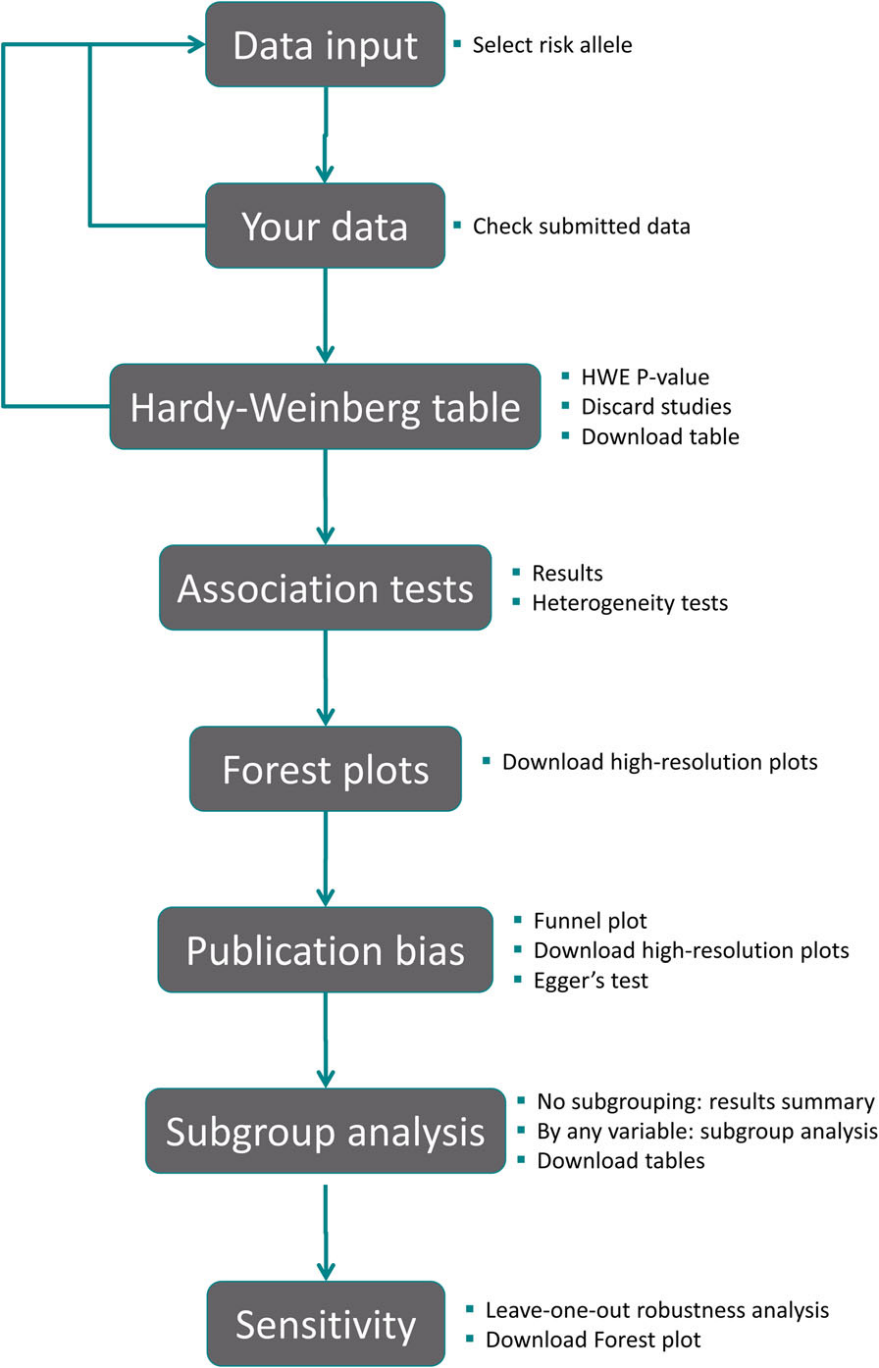
Overview of MetaGenyo. The scheme represents the tool’s workflow. First, data is uploaded by the user and it can be reviewed. Secondly, HWE *P*-values are calculated, so users can decide to exclude some bad-quality samples and reupload their data. In Association tests, Forest plots, Publication bias and Subgroup analysis tabs users can download the meta-analysis results. Finally, users can check the sensitivity analysis

An overview of MetaGenyo is provided in the on-line help of the application and Fig. 1. First, the user loads the collected data from individual studies as a text or Excel file with some specifications on the file format. Once the data has been loaded, a complete analysis is performed providing results and visualizations in different tabs: (1) The data tab, where the user can check if the data has been correctly submitted. (2) Hardy-Weinberg tab, where a HWE *P*-value column is added to the data. (3) Association values tab. This contains different association values and heterogeneity indicators for each genetic model. (4) Forest plot tab contains forest plot visualizations in high-quality image format for each genetic model. (5) Publication bias tab, where the user can see the funnel plot and Egger’s test results. (6) Subgroup analysis tab to obtain a summary of the analysis or to evaluate the association and heterogeneity results taking into account stratification based on user-defined variables and, finally, (7) Sensitivity tab to perform a robustness analysis.

## Results and discussion

Despite there are many programs designed to perform genome-wide association studies (GWAS) meta-analysis (reviewed in [19]), there is a lack of tools specially designed to perform GAS meta-analysis, so researchers use general statistical or meta-analysis software, adapting it to the particular purposes in such type of meta-analysis. This lack of dedicated software increases the required resources to perform a GAS meta-analysis, facilitates the inclusion of methodological errors and requires advanced bioinformatics expertise.

Among the most widely used software solutions in this field are STATA [5], SPSS [6] and SAS [20]. These are popular software suites that provide a set of statistical functions that can be used in a broad range of applications and data analysis problems, but they are proprietary software and are not specialized in GAS meta-analysis. These limitations are partially overcome by R packages such as meta [7], rmeta [21] and metafor [8]. These are freely-available software libraries to perform a complete meta-analysis in a flexible way. However, their use requires R programming skills, they do not provide a guided workflow and they are not specifically designed to perform GAS meta-analysis. In addition, there are some Excel extensions such as MIX [22] and MetaEasy [23]. These extensions are easy to use, but they require the usage of the proprietary software Microsoft Excel.

In this context, MetaGenyo is a user-friendly web application that implements a complete meta-analysis following a guided workflow, which does not require programming knowledge. Table 1 contains a summary of the main advantages and disadvantages of some reviewed GAS meta-analysis software.

**Table 1:** Characteristics of available meta-analysis software

	STATA	SPSS	MIX	MetaEasy	meta	rmeta	metafor	MetaGenyo
USABILITY								
Availability	Commercial	Commercial	Commercial^a^	Free^b^	Free	Free	Free	Free
Web-based	No	No	No	No	No	No	No	Yes
Operating system	Windows, Mac OS, Linux	Windows, Mac OS, Linux	Windows	Windows	Windows, Mac OS, Linux	Windows, Mac OS, Linux	Windows, Mac OS, Linux	Any^c^
Guided workflow	No	No	No	No	No	No	No	Yes
Programming knowledge	Yes^d^	Yes^d^	No	No	R language	R language	R language	No
FUNCTIONALITIES								
Specific for GAS meta-analysis	No	No	No	No	No	No	No	Yes
HWE testing	Yes	No	No	No	No	No	No	Yes
Heterogeneity assessment	Yes	Yes	Yes	Yes	Yes	Yes	Yes	Yes
Random/Fixed effect models	Yes	Yes	Yes	Yes	Yes	Yes	Yes	Yes
Forest plot	Yes	Yes	Yes	Yes	Yes	Yes	Yes	Yes
Automatic testing of genetic models	No	No	No	No	No	No	No	Yes
Publication bias	Yes	Yes	Yes	No	Yes	Yes	Yes	Yes
Subgroup analysis	Yes	No	Yes	No	Yes	No	Yes	Yes
Robustness analysis	Yes	No	Yes	No	Yes	No	Yes	Yes
P-value correction for multiple testing	Yes	Yes	No	No	No	No	No	Yes

^a^There is a MIX free version with reduced capabilities. ^b^MetaEasy is free, but it depends on the proprietary software Microsoft Excel. ^c^MetaGenyo is accessed through an internet browser, so there are no limitations regarding the operating system used to access it. ^d^Although STATA and SPSS are command-based software, there are graphical user interfaces (GUIs) available which permits replacing scripting by user-friendly interactive commands

To demonstrate the functionality of MetaGenyo we have used data from a published GAS meta-analysis [24]. In this study, the authors performed a meta-analysis to study the association between the A23G SNP of XPA gene (rs1800975) and digestive cancers. They collected genotype information from 18 case-control studies including 4170 patients and 6929 controls in total. In this polymorphism, the G allele was considered the reference, so the A allele was the risk allele (this parameter must be specified in MetaGenyo). Results from the complete analysis and a comparison with results reported in the original article can be found in Additional file 1.

Briefly, both sets of results are highly concordant, but in the original publication the authors did not correct the *P*-values for multiple testing or evaluated different genetic models as provided by MetaGenyo. We found some discrepancies between both sets of results due to use of inappropriate statistical tests or labeling mistakes, especially at the subgroup analysis step (see Additional file 1). Because MetaGenyo automatically performs all meta-analysis steps in a guided analysis we reduced these potential sources of errors. All these similarities and differences are detailed in Additional file 1.

**Fig. 2:**
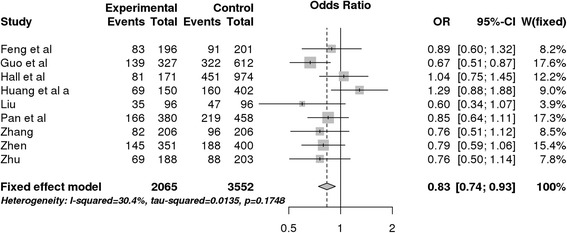
Forest plot of esophageal cancer data generated with MetaGenyo. The tested comparison is AG vs. AA + AG (overdominant model) and FEM was used

The application generated results for all possible genetic models and allowed us to easily evaluate results for different subgroups in a unified framework. In this context, using the tumor type feature to stratify the data revealed a significant association for the overdominant model in esophageal cancer studies not previously reported (OR = 0.83, 95% CI = 0.74–0.93, *P*-value = 0.0016, Bonferroni-adjusted P-value = 0.0448) [Fig. 2]. Although the original work reported no significant association between this polymorphism and the risk of any type of digestive cancer for the studied models, there may be a protective effect of AG genotype against the risk of esophageal tumors overlooked at the original article because the authors did not test this genetic model. Indeed, a similar association has been found in another GAS meta-analysis with lung cancer samples [25].

## Conclusions

In this work, we present MetaGenyo, a free easy-to-use web tool to perform GAS meta-analysis. It provides a guided workflow through the most important steps of a meta-analysis.

We demonstrated MetaGenyo’s functionality replicating a previously published meta-analysis [24]. In addition, thanks to the automatic testing of several genetic models and subgroup analysis we found a significant association between rs1800975 SNP in XPA gene and esophageal cancer under the overdominant genetic model that may be interesting enough for further testing.

Surprisingly, there is a large heterogeneity in statistical methods, lack of quality control steps or misleading reporting and interpretation of results in many published meta-analysis [9]. Therefore, an application such as MetaGenyo will be a very useful tool for the research community providing a guided and solid workflow.

### Availability

Project name: MetaGenyo.

Availability: MetaGenyo web tool, example datasets and help are accessible at http://bioinfo.genyo.es/metagenyo/.

Any restrictions on use by academics: none.

## Additional file

Additional file 1: MetaGenyo’s use case. Document showing the results of analyzing the data provided by [24] using MetaGenyo and comparison with the original results. (PDF 253 kb)

## Abbreviations

CI: Confidence intervals
FDR: False discovery rate
FEM: Fixed effect model
GAS: Genetic association study
GUI: Graphical user interface
GWAS: Genome-wide association study
HWE: Hardy-Weinberg equilibrium
OR: Odds-ratio
REM: Random effect model
χ^2^: Goodness-of-fit chi-square
