# Supplementary material for: MetaGenyo: a web tool for meta-analysis of genetic association studies
Source: BMC Bioinformatics. 2017 Dec 16;18:563. doi: 10.1186/s12859-017-1990-4 (PMC5732412; doi:10.1186/s12859-017-1990-4)
Supplement: Additional file 1: — MetaGenyo’s use case. Document showing the results of analyzing the data provided by [24] using MetaGenyo and comparison with the original results. (PDF 253 kb) [file 12859_2017_1990_MOESM1_ESM.pdf]

Additional file 1

Here we provide a use case and a detailed analysis of results from the reanalysis of data reported by He et al. [1].

The input file for MetaGenyo was a text file containing the original data of the meta-analysis and reformatted to accomplish with the MetaGenyo input format [Supplementary table 1].

Supplementary table 1. Input table for the example meta-analysis.

Author	Ethnicity	Tumor type	Source of control	GG cases	GA cases	AA cases	GG control	GA control	AA control
**Dong et al**	Asian	Gastric	PB	47	120	86	128	322	162
**Feng et al**	Asian	Esophageal	HB	28	83	85	56	91	54
**Gil et al**	Caucasian	Colorectal	HB	26	58	16	50	67	16
**Guo et al**	Asian	Esophageal	PB	65	139	123	128	322	162
**Hall et al**	Caucasian	Esophageal	HB	75	81	15	398	451	125
**Hansen et al**	Caucasian	Colorectal	PB	176	187	31	339	359	90
**Huang et al a**	Asian	Esophageal	PB	22	69	59	32	160	210
**Huang et al b**	Asian	Cardiac	PB	20	60	65	13	55	112
**Huang et al c**	Asian	Gastric	PB	12	57	77	13	55	112
**Jelonek et al**	Caucasian	Colorectal	HB	29	33	4	46	70	17
**Joshi et al**	Caucasian	Colorectal	PB	136	133	33	149	170	30
**Liu**	Asian	Esophageal	PB	11	35	50	11	47	38
**Palli et al**	Caucasian	Gastric	PB	134	115	35	249	215	59
**Pan et al**	Caucasian	Esophageal	HB	179	166	35	151	219	88
**Xie**	Asian	Hepatocellular	PB	139	203	73	144	219	116
**Zhang**	Asian	Esophageal	HB	33	82	91	44	96	66
**Zhen**	Asian	Esophageal	PB	99	145	107	53	188	159
**Zhu**	Asian	Esophageal	PB	50	69	69	52	88	63

Abbreviations: PB=population-based; HB=hospital-based.

The application guides the user across the statistical functions that should be used in the analysis, allowing non-expert users to perform a complete meta-analysis covering all the required steps.

P-values for HWE were calculated in controls and adjusted P-values greater than 0.05 indicated that the study fits with HWE conditions [Supplementary table 2]. The unadjusted P-values are the same that those calculated by the original authors [Table 1 in the original paper]. However, as the analysis comprises several studies, it is important to adjust P-values for multiple testing in order to reduce false positives, which were not calculated by the original authors. MetaGenyo corrects P-values by FDR method.

Supplementary table 2. P-value and adjusted P-value by FDR of χ^2 test for HWE in control samples of each study.

Author	HWE P-value	HWE adjusted P-value
**Dong et al**	0.1694	0.4064
**Feng et al**	0.1806	0.4064
**Gil et al**	0.3686	0.6032
**Guo et al**	0.1694	0.4064
**Hall et al**	0.8751	0.8751
**Hansen et al**	0.7312	0.8751

**Huang et al a**	0.8434	0.8751
**Huang et al b**	0.0966	0.3478
**Huang et al c**	0.0966	0.3478
**Jelonek et al**	0.2252	0.4088
**Joshi et al**	0.0558	0.3478
**Liu**	0.5353	0.7412
**Palli et al**	0.2271	0.4088
**Pan et al**	0.5893	0.7577
**Xie**	0.0711	0.3478
**Zhang**	0.4116	0.6174
**Zhen**	0.8259	0.8751
**Zhu**	0.0631	0.3478

In the next step, statistical associations were evaluated for different genetics models. In the original work, the authors evaluated four different genetic models: dominant model (AA + AG vs. GG), recessive model (AA vs. AG + GG), heterozygote comparison (AG vs. GG) and homozygote comparison (AA vs. GG). All those comparisons can be performed with MetaGenyo, in addition to allele contrast (A vs. G), overdominant model (AG vs. AA + GG) and (AA vs. AG) comparison. A forest plot was obtained to summarize the results applying REM statistics with dominant genetic model [Supplementary figure 1], as the original authors did [Figure 2 in the original paper]. We noticed that some individual statistics are slightly different between the original forest plot and those reported by MetaGenyo. After comparing the forest plot reported in the original publication and the data that was used to generate it, we realized that some labels were exchanged in this plot (e.g. Guo et al. label actually contains the data from Gil et al. study). This mislabeling caused the discrepancies between both forest plots.

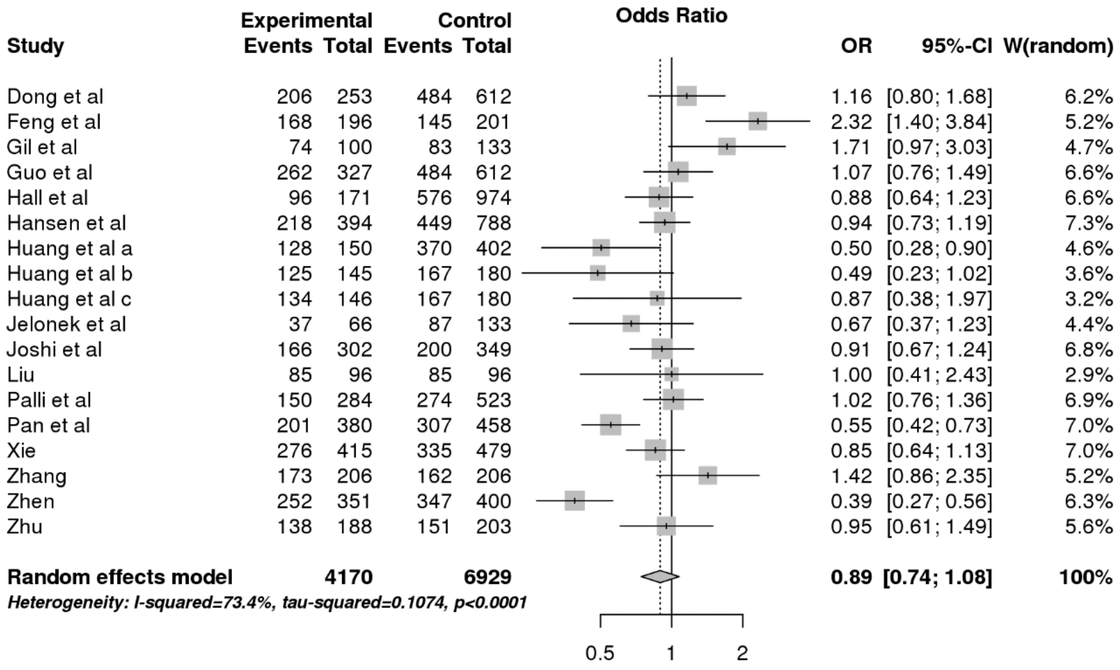

Supplementary figure 1. Forest plot with dominant genetic model and REM statistics generated with MetaGenyo.

For subgroup analysis, the original authors reported the results using forest plots [Figures 3 and 4 in the original paper]. MetaGenyo performs subgroup analysis generating a summary table with the selected factor [Supplementary tables 2 and 3]. All results reported with MetaGenyo with data stratified by ethnicity match the original results. However, for the meta-analysis stratified by tumor type, results differ for colorectal and gastric tumors. We realized that the original authors applied REM statistics for these groups. However, the heterogeneity indicators show that, for these subgroups, there is not significant heterogeneity ($I^2 < 50$ % and heterogeneity P-values > 0.1), so FEM should be used instead of REM [2]. For subgroup analysis, MetaGenyo applies REM or FEM statistics depending on the heterogeneity P-value (REM if P-value < 0.1, FEM if otherwise). We repeated the meta-analysis for both, colorectal and gastric tumors, and we obtained the same results as the authors of the original work for the former (data not shown).

However, in gastric tumor samples in which we did not found agreement, we observed that, for some reason, original authors included samples from cardiac cancer in the gastric cancer group, causing these discrepancies with MetaGenyo results.

Supplementary table 2. MetaGenyo's subgroup analysis results with dominant genetic model and splitting the samples by ethnicity.

Ethnicity	Test of association			Test of heterogeneity		
	**OR**	**95 % CI**	**P-value**	**Model**	I^2	**P-value**
Overall	0.8940	[0.7426; 1.0762]	0.2365	Random	0.7339	0.0001
Asian	0.8968	[0.6625; 1.2139]	0.4807	Random	0.7823	0.0001
Caucasian	0.8809	[0.7059; 1.0993]	0.2619	Random	0.6626	0.0068

Supplementary table 3. MetaGenyo's subgroup analysis results with dominant genetic model and splitting the samples by tumor type.

Tumor type	Test of association			Test of heterogeneity		
	**OR**	**95 % CI**	**P-value**	**Model**	I^2	**P-value**
Overall	0.8940	[0.7426; 1.0762]	0.2365	Random	0.7339	0.0001
Colorectal	0.9549	[0.8025; 1.1362]	0.6029	Fixed	0.4521	0.1401
Esophageal	0.8668	[0.6102; 1.2314]	0.4249	Random	0.8395	0.0001
Gastric	1.0527	[0.8449; 1.3117]	0.6471	Fixed	0.0000	0.7701

A funnel plot was also generated, revealing that there was not publication bias in the data (see Supplementary figure 2). This MetaGenyo output is very similar to the previously published one [Figure 5 in the original paper], except the x and y axes are the opposite between both figures and MetaGenyo use the OR as the x axis, while the original plot uses the log(OR).

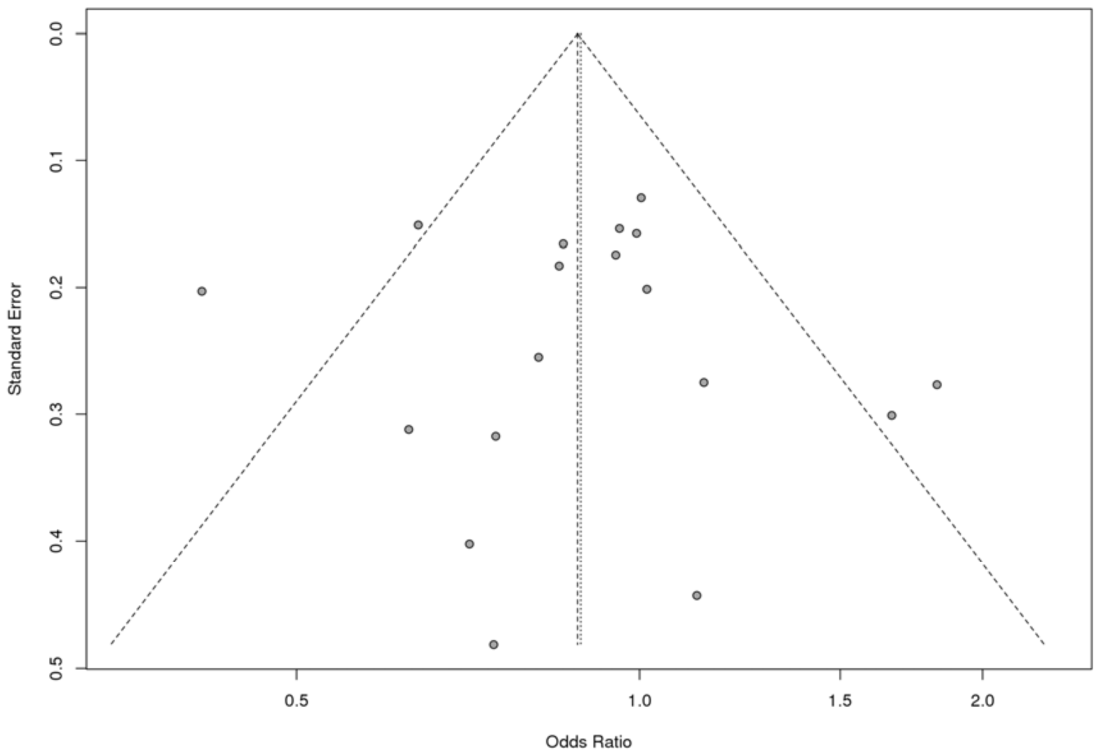

Supplementary figure 2. Funnel plot of AG vs. GG comparison generated by MetaGenyo.

Finally, a sensitivity analysis was performed with MetaGenyo generating a forest plot of the results excluding one of the studies in each step [Supplementary figure 3] revealing that the results were not biased by any single study from those originally included in the work. The original authors performed the same sensitivity analysis and reached the same conclusions, but they did not include a forest plot of such analysis.

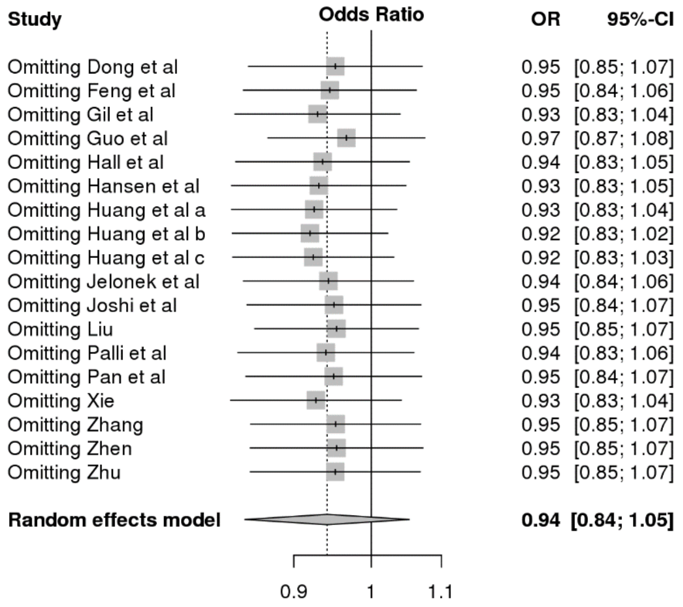

Supplementary figure 3. Forest plot of sensitivity analysis under overdominant model and REM statistics.

References

1. He L, Deng T, Luo H. XPA A23G polymorphism and risk of digestive system cancers: a meta-analysis. Onco Targets Ther. 2015;8:385–94.

2. Ried K. Interpreting and understanding meta-analysis graphs--a practical guide. Aust Fam Physician. 2006;35(8):635–8.
